# Exploring burnout among psychiatric trainees at a South African university

**DOI:** 10.4102/sajpsychiatry.v28i0.1634

**Published:** 2022-02-25

**Authors:** Tejil Morar, Belinda Marais

**Affiliations:** 1 Department of Psychiatry, Faculty of Health Sciences, University of the Witwatersrand, Johannesburg, South Africa; 2 Gauteng Department of Health, Sterkfontein Hospital, Johannesburg, South Africa; 3 Gauteng Department of Health, Tara Hospital, Johannesburg, South Africa

**Keywords:** burnout, doctors’ mental health, psychiatry, trainees, stress, South African doctors, Maslach Burnout Inventory, residents

## Abstract

**Background:**

The mental health of doctors is increasingly topical, internationally and locally. Of importance is the phenomenon of burnout, a far-reaching repercussion of chronic work-related stress. Psychiatrists are more vulnerable to stress, burnout and suicide in comparison with other medical specialities. There is a void in published research relating to South African psychiatric trainees.

**Aim:**

The study aimed to investigate burnout and associated factors among psychiatric registrars at a South African university.

**Setting:**

Department of Psychiatry at the University of the Witwatersrand.

**Methods:**

This was a cross-sectional study via an anonymous self-administered questionnaire. The questionnaire comprised three sections: demographics; the Maslach Burnout Inventory Human Services Survey (MBI-HSS) and questions relating to contributing factors, protective factors and consequences of burnout. The MBI-HSS is recognised as the leading measure of burnout, consisting of three subscales: emotional exhaustion (EE), depersonalisation (DP) and personal accomplishment (PA).

**Results:**

The questionnaire was completed by 33 out of 55 psychiatric registrars (60.0% response rate). Data from 31 registrars were used in the analyses, as two registrars did not provide informed consent. Among participants, EE was the most commonly affected, followed by DP and lastly PA. The majority (67.8% or *n* = 21) had scores in the high category for any one of the three subscales (EE/DP/PA). Significant factors associated with burnout included poor work and non-professional life balance (*p* = 0.017), utilising annual leave days for work-related tasks (*p* < 0.001), irregular holidays (*p* = 0.003) and financial debt (*p* = 0.026). A possible protective factor was an amicable relationship with fellow psychiatric registrars.

**Conclusion:**

There is evidence of some degree of burnout in more than two-thirds of participants. Associated factors lie largely at an organisational level, and while optimising individual resilience is important, systemic support plays a key role.

## Introduction

The mental health of doctors is increasingly topical, both internationally and locally. Of importance is the phenomenon of burnout, a far-reaching repercussion of chronic work-related stress.^1^ Psychiatrists are more vulnerable to stress, burnout and suicide in comparison with other medical specialities.^1,2^ There is a void in published research relating to South African psychiatric trainees.

American psychoanalyst Herbert Freudenberger coined the term *burnout* in 1974.^3^ The Diagnostic and Statistical Manual of Mental Disorders, fifth edition (DSM-5) does not acknowledge burnout as a diagnosis or an adequately valid clinical entity.^4^ However, the phenomenon is increasingly recognised in research as it is regarded as impairing and consequential. The 10th revision of the International Statistical Classification of Diseases and Related Health Problems (ICD) recognises burnout (Z73.0) under problems related to life-management difficulty.^5^ In May 2019, the World Health Organization (WHO) revealed that burnout would be defined in the ICD-11 as follows:

[*A*] syndrome conceptualized as resulting from chronic workplace stress that has not been successfully managed. It is characterized by three dimensions: feelings of energy depletion or exhaustion; increased mental distance from one’s job, or feelings of negativism or cynicism related to one’s job; and reduced professional efficacy. Burn-out refers specifically to phenomena in the occupational context and should not be applied to describe experiences in other areas of life.^6^

Various instruments have been developed to measure burnout, including the Maslach Burnout Inventory (MBI), the Oldenburg Burnout Inventory, the Physician Work-Life Study’s single item and the Copenhagen Burnout Inventory.^7^ The MBI is recognised as the leading measure of burnout with the strongest set of psychometric properties. The MBI Human Services Survey for Medical Personnel [MBI-HSS (MP)] has been developed for professionals working with patients. It comprises three subscales: emotional exhaustion (EE) – the central quality of burnout, depersonalisation (DP) and professional accomplishment (PA).^8^

The burnout rate among doctors varies from 17.6% – 82.0%, subject to speciality, setting, methods and subscales measured.^9^ Among psychiatrists, Umene-Nakano et al. found that 21.0% of respondents had high levels of EE, 12.0% had high levels of DP and 72.0% had low levels of PA.^10^ In the largest study on burnout among psychiatric trainees, to date, Jovanović et al. obtained information from 22 countries (including South Africa), and severe burnout was found in 36.7% of the participants.^11^

The current literature describes internal and external contributing factors to burnout. Internal factors refer to individual and personality-related factors, while external factors include the environment and situational influences.^12^

Regarding individual factors, age is the demographic variable most consistently related to burnout. Maslach et al. found that younger employees (< 40 years) and single individuals are more prone.^13^ Race and the burden of *black tax* may play a role. It has been shown that even when minorities achieve faculty rank, they report lower career satisfaction and higher social isolation.^14^ The concept of *black tax* is recognised in African ethnic populations and refers to the financial obligation experienced by black professionals to support their extended families.^15^ Furthermore, Semmer described the inherent personality profile of a stress-prone individual: poor levels of resilience, low self-esteem, an external locus of control and an avoidant coping style.^16^

External contributing factors were identified by Fothergill et al. in a systematic review: patient and family negative characteristics, administrative and organisational inadequacies, lack of resources, staff conflicts, lack of encouraging feedback, disproportionate supervisory duties, long working hours, excessive workload, violence and suicide perpetrated by patients, low pay and role conflict (between work and personal life).^17^ Gouveia et al. identified that fear of making mistakes, duality of roles, competitiveness and lack of autonomy play a role.^18^

In low- and middle-income countries, psychiatrists are scarce and disproportionately distributed.^19^ In South Africa, poor working conditions in the state sector predispose doctors to burnout, as doctors work strenuous overtime hours and frequently face crisis situations and large patient volumes.^20,21^ Policymakers are often at a provincial or national level, leaving doctors alienated from decisions having a major impact on themselves.^22^

Moderators of burnout, described by Fothergill et al., include supportive personal relationships (community support, relationships with family and friends and private therapy), attention to non-professional life, regular holidays and designated time for medical training.^17^ Furthermore, Fischer et al. noted factors protective against burnout: supportive professional relationships, variety in the workplace, time at work allocated for non-clinical duties and a positive attitude with high job satisfaction.^23^

The consequences of burnout extend from personal to healthcare system outcomes. Personal outcomes of burnout include depression, adverse effects on relationships, substance abuse, physical illness and increased suicide risk.^17^ One in 10 doctors in Cape Town reportedly prescribe antidepressants for their personal use.^24^ Among physicians, 8.0% – 12.0% are predicted to develop a substance use disorder at some point in their career.^25^ Physician suicide rates are approximately six times higher than in the general population, and the greatest suicidal tendencies among male physicians occur among psychiatrists.^17,25^ The WHO highlighted that work stress contributes to cardiovascular, gastrointestinal and musculoskeletal disorders.^26^ Burnout alters neural circuits, specifically the amygdala, and has been shown to result in cortical thinning, decreased fine motor function and the inability to shift attention. Doctors develop maladaptive coping mechanisms in response to burnout, such as emotional detachment, which may be especially detrimental in psychiatry.^27^

The outcomes of burnout in healthcare systems are recruitment and retention issues, lowered productivity and efficiency (including absenteeism), suboptimum patient care, medical errors, higher medical aid expenditure and decreased patient satisfaction.^25,26^ Burnout has been identified as a cause of medical migration in South Africa, and failure to address it may result in the loss of skills to the global labour market.^21^ The Gauteng Department of Health and Social Development faced malpractice claims worth R573 million in 2009–2010.^28^ The Health Professions Council of South Africa (HPCSA) found 90 doctors guilty of unprofessional conduct in one year.^28^ While many factors play a role in malpractice and unprofessional conduct, staff well-being may contribute.^25^

The former president of the South African Society of Psychiatrists, Professor Janse van Rensburg, highlighted the responsibility, at an organisational level, to recognise burnout as a problem.^29^

## Aim and objectives

This research study aimed to investigate burnout among psychiatric registrars (trainees) at the University of the Witwatersrand.

The study objectives were as follows:

- to determine the scores of the three subscales of burnout, EE, DP and PA, using the MBI-HSS (MP)
- to ascertain the demographic and work-related profiles of participants and their associations with MBI-HSS (MP) scores
- to ascertain potential contributing and protective factors, as well as potential consequences of burnout.

## Research methods and design

### Study setting

The study participants were registrars in the Department of Psychiatry at the University of the Witwatersrand (WITS), South Africa.

### Study design

This study was a cross-sectional investigation of burnout and its associated factors.

### Study population and sampling strategy

There were 55 registrars in the WITS Department of Psychiatry at the time of the study.^30^ All registrars who provided informed consent to participate in the study were included. In order to ensure anonymity, unidentifiable questionnaires were distributed and returned, via the WITS Department of Psychiatry’s internal mail, to the department secretary in October 2018.

### Data collection and instrument

The questionnaires (based on a literature review) were self-administered by the participants and comprised of three sections:

- demographics and work-related variables
- the MBI-HSS (MP)
- contributing factors, protective factors and possible consequences of burnout.

Permission to use the MBI instrument was obtained, and copies thereof purchased from www.mindgarden.com. Human services are divided into social service, medical, mental health and other.^8^ This tool has been administered over a variety of countries, languages and cultural backgrounds.^8^ The reliability of the MBI-HSS (MP) has been estimated using Cronbach’s coefficient alpha to be 0.90 for EE, 0.79 for DP and 0.71 for PA. Validity for the MBI-HSS has been confirmed by various studies and meta-analyses.^8^

### Data analysis

Data were analysed using Microsoft^TM^ Excel and STATISTICA 7.1 statistical software (www.statsoft.com). The dataset did not meet the assumptions of normality; therefore, non-parametric analyses were used. All statistical tests were two-tailed, and alpha was set at 0.05.

Objective 1: Descriptive statistics for EE, DP and PA were generated. Scores were categorised as high based on a threshold as per the cut-off score published in the third edition of the MBI manual^8^ (Table 1). These cut-off scores correlate with the scores predominantly used in burnout research.^31^ The higher the EE and DP score and the lower the PA score, the more likely the individual is to experience burnout.^8^

**TABLE 1:** Classification of burnout.

Category	Low	Average	High
EE (9 questions)	≤ 18	19–26	≥ 27
DP (5 questions)	≤ 5	6–9	≥ 10
PA (8 questions)	≥ 40	39–34	≤ 33

*Source*: Doulougeri K, Georganta K, Montgomery A. “Diagnosing” burnout among healthcare professionals: Can we find consensus? Cogent Med. 2016;3(1):Article 1. https://doi.org/10.1080/2331205X.2016.1237605

EE, emotional exhaustion; DP, depersonalisation; PA, personal accomplishment.

Objective 2: Demographics and work-related profiles of participants were tabulated. The Mann-Whitney U and Kruskal–Wallis tests were used to analyse significant associations between participants’ demographic and work-related profiles and their MBI results.

Objective 3: Contributing, protective factors and possible consequences of burnout were analysed using the Mann–Whitney U and Kruskal–Wallis tests.

### Ethical considerations

Permission to conduct the study was obtained from the Deputy Registrar of WITS, WITS Head of Department of Psychiatry and the WITS Human Research Ethics Committee (Medical) – clearance number M180556. A distress protocol was included in the questionnaire.

## Results

Out of 55 psychiatric registrars, 33 completed and returned the questionnaire (a 60.0% response rate). Data from 31 registrars were used in the analyses, as two registrars did not provide informed consent.

### Objective 1

Among the participants, EE was the most commonly affected, followed by DP and PA (Table 2). The majority (67.7% or *n* = 21) had scores in the high category for any one of the three subscales (EE/DP/PA).

**TABLE 2:** Descriptive statistics and scores of the Maslach Burnout Inventory subscales among psychiatric registrars at University of the Witwatersrand.

Subscale	EE		DP		PA	
	%	*n*	%	*n*	%	*n*
Mode	39	-	2	-	5.29	-
Range	10–46	-	0–23	-	21–47	-
Low	16.1	5	41.9	13	16.1	5
Average	32.2	10	19.4	6	48.4	15
High	51.6	16	38.7	12	35.5	11

EE, emotional exhaustion; DP, depersonalisation, PA, personal accomplishment.

### Objective 2

None of the demographic factors (Table 3) or work-related profiles (Table 4) were significant predictors for the EE, DP and PA subscales.

**TABLE 3:** Demographics and Maslach Burnout Inventory subscale scores.

Variable	Population distribution		Sample distribution†		EE						DP						PA					
	*n*	%	*n*	%	Median	First IQR	Third IQR	*U*	*p*	*X* ^2^_2_	Median	First IQR	Third IQR	*U*	*p*	*X* ^2^_2_	Median	First IQR	Third IQR	*U*	*p*	*X* ^2^_2_
**Gender**	-	-	-	-	-	-	-	48.5	0.223	-	-	-	-	12.5	0.070	-	-	-	-	46.0	0.178	
Female	44	80.0	24	77.4	23.8	13.0	30.0	-	-	-	3.8	0.0	7.5	-	-	-	35.0	32.5	38.0	-	-	-
Male	11	20.0	6	19.4	20.5	10.0	23.5	-	-	-	3.5	2.0	12.0	-	-	-	39.0	35.5	41.8	-	-	-
**Age**	Unknown		-	-	-	-	-	58.5	0.409	-	-	-	-	56.5	0.355	-	-	-	-	71.5	0.861	-
< 35 years	-	-	25	80.6	20.0	10.0	26.0	-	-	-	7.00	4.0	16.0	-	-	-	31.0	21.0	35.0	-	-	-
> 35 years	-	-	6	19.4	26.3	22.0	33.0	-	-	-	4.5	2.5	7.3	-	-	-	35.0	29.0	36.0	-	-	-
**Race**	-	-	-	-	-	-	-	-	0.264	2.6	-	-	-	-	0.343	-	-	-	-	-	0.593	1.0
Black	32	58.2	13	41.9	32.0	25.0	35.0	-	-	-	7.0	4.0	10.0	-	-	-	35.0	31.0	38.0	-	-	-
Indian/Asian	12	21.8	8	41.9	24.0	17.5	31.5	-	-	-	10.0	3.0	16.0	-	-	-	37.0	29.8	39.3	-	-	-
White	11	20.0	8	25.8	27.5	19.5	38.3	-	-	-	9.0	4.3	11.0	-	-	-	34.5	33.0	37.3	-	-	-
**Religion or spirituality**	-	-	-	-	-	-	-	-	0.337	3.4	-	-	-	-	0.473	2.5	-	-	-	-	0.745	1.2
Religious	Unknown		11	35.5	25.0	23.0	34.0	-	-	-	9.0	3.0	13.0	-	-	-	35.0	31.5	37.0	-	-	-
Spiritual	-	-	7	22.6	31.0	29.0	39.0	-	-	-	13.0	4.5	16.0	-	-	-	33.0	30.5	38.5	-	-	-
Both	-	-	10	32.3	29.0	19.5	36.5	-	-	-	7.5	3.0	12.8	-	-	-	37.5	33.5	38.0	-	-	-
Neither	-	-	22	71.0	18.0	15.5	20.5	-	-	-	4.5	3.3	5.8	-	-	-	32.5	30.3	34.8	-	-	-
**Relationship status**	-	-	-	-	-	-	-	-	0.737	0.6	-	-	-	-	0.837	0.4	-	-	-	-	0.447	1.6
Married	Unknown		16	51.6	28.0	24.8	33.5	-	-	-	8.5	4.8	11.0	-	-	-	35.0	32.5	37.3	-	-	-
Relationship	-	-	8	25.8	22.5	19.8	32.3	-	-	-	5.0	2.0	16.5	-	-	-	37.0	33.3	40.3	-	-	-
Single	-	-	7	22.6	31.0	22.5	39.0	-	-	-	5.0	4.0	14.5	-	-	-	37.0	32.0	38.0	-	-	-
**Children**	-	-	-	-	-	-	-	98.5	0.709	-	-	-	-	77.5	0.245	-	-	-	-	93.5	0.645	-
Yes	Unknown		16	51.6	32.0	25.0	35.0	-	-	-	7.0	4.0	9.0	-	-	-	35.0	31.0	37.0	-	-	-
No	-	-	13	41.9	29.0	19.8	38.3	-	-	-	13.5	3.0	16.0	-	-	-	36.0	32.3	38.0	-	-	-
**Supporting extended family**	-	-	-	-	-	-	-	105	0.553	-	-	-	-	75.5	0.079	-	-	-	-	88.5	0.213	-
Yes	Unknown		15	48.4	30.0	23.0	36.5	-	-	-	5.0	2.5	8.5	-	-	-	37.0	35.0	37.0	-	-	-
No	-	-	16	51.6	26.5	19.8	33.8	-	-	-	11.5	6.3	16.0	-	-	-	33.5	29.8	38.0	-	-	-

EE, emotional exhaustion; DP, depersonalisation; PA, personal accomplishment; IQR, interquartile range.

† , In cases where *n* ≠ 31, the remainder of participants chose not to answer the question.

**TABLE 4:** Work-related profiles and Maslach Burnout Inventory subscale scores.

Variable	Population distribution		Sample distribution†		EE					DP					PA				
	*n*	%	*n*	%	Median	First IQR	Third IQR	*p*	*X* ^2^_2_	Median	First IQR	Third IQR	*p*	*X* ^2^_2_	Median	First IQR	Third IQR	*p*	*X* ^2^_2_
**Duration of registrar training**	-	-	-	-	-	-	-	0.396	3	-	-	-	0.397	3.1	-	-	-	0.560	2.9
< 12 months	8	14.5	6	19.4	29.0	25.0	36.0	-	-	5.5	2.0	12.0	-	-	34.0	30.8	38.8	-	-
12-36 months	26	47.2	18	58.1	28.0	20.5	34.5	-	-	7.5	4.0	16.0	-	-	36.0	33.0	38.0	-	-
> 36 months	21	38.1	6	19.4	29.0	22.0	37.5	-	-	6.5	5.0	8.8	-	-	36.0	30.5	41.5	-	-
**Most challenging rotation**	-	-	-	-	-	-	-	0.566	2.9	-	-	-	0.434	3.8	-	-	-	0.566	3
Child and adolescent	Unknown		4	12.9	28.5	23.5	33.8	-	-	6.0	4.5	9.3	-	-	40.0	35.5	44.0	-	-
Community	-	-	7	22.6	30.0	26.5	34.0	-	-	13.0	9.0	17.0	-	-	34.0	31.5	37.0	-	-
Forensics	-	-	6	19.4	24.5	19.3	29.8	-	-	7.5	3.3	8.8	-	-	35.0	29.3	37.8	-	-
General psychiatry	-	-	4	12.9	26.5	17.5	34.5	-	-	10.0	3.5	16.5	-	-	35.0	31.5	36.8	-	-
Neorology/neuropsychiatry	-	-	1	3.2	21.0	21.0	21.0	-	-	5.0	5.0	5.0	-	-	35.0	35.0	35.0	-	-
**Pending exams**	-	-	-	-	-	-	-	0.678	0.8	-	-	-	0.280	2.5	-	-	-	0.383	1.9
None	Unknown		18	58.1	28.0	23.3	38.0	-	-	15.5	8.5	23.0	-	-	36.0	33.0	37.8	-	-
Part I	-	-	7	22.6	30.0	24.0	35.0	-	-	14.5	9.0	18.0	-	-	33.0	30.5	37.5	-	-
Part II	-	-	6	19.4	23.5	21.3	31.0	-	-	7.3	5.0	16.0	-	-	39.0	35.5	42.5	-	-

EE, emotional exhaustion; DP, depersonalisation; PA, personal accomplishment.

† , In cases where *n* ≠ 31, the remainder of participants chose not to answer the question.

### Objective 3

Factors associated with higher EE scores were as follows (Table 5):

- poor work and non-professional life balance
- utilising leave days for work-related tasks
- not taking regular holidays
- debt
- attending therapy.

**TABLE 5:** The relationship between contributing and protective factors and Maslach Burnout Inventory subscale scores.

Variable	Categories†		EE						DP						PA					
	*n*	%	Median	First IQR	Third IQR	*U*	*p*	*X* ^2^_2_	Median	First IQR	Third IQR	*U*	*p*	*X* ^2^_2_	Median	First IQR	Third IQR	*U*	*p*	*X* ^2^_2_
**Able to delegate tasks**	-	-	-	-	-	-	0.235	2.39	-	-	-	-	0.796	0.5	-	-	-	-	0.438	1.7
Yes	13	41.9	25.0	23.0	32.0	-	-	-	8.0	3.0	13.0	-	-	-	37.0	34.0	38.0	-	-	-
No	12	38.7	30.0	20.8	39.0	-	-	-	8.5	5.0	14.5	-	-	-	35.0	28.8	37.0	-	-	-
Unsure	6	19.4	32.0	23.0	37.5	-	-	-	4.0	4.0	13.0	-	-	-	34.0	31.5	38.0	-	-	-
**Balance between work and non-professional life**			-	-	-	-	0.017*	10.2	-	-	-		0.730	1.3	-	-	-	-	0.566	4
Yes	14	45.2	23.0	19.3	27.3	-	-	-	2.3	2.0	9.8	-	-	-	31.5	33.3	40.3	-	-	-
No	11	35.5	33.0	30.0	39.5	-	-	-	4.0	4.5	17.0	-	-	-	31.0	31.0	37.5	-	-	-
Unsure	5	16.1	26.0	25.0	31.0	-	-	-	8.0	6.3	16.0	-	-	-	37.0	34.3	38.0	-	-	-
**Self-care**	-	-	-	-	-	-	0.122	4.2	-	-	-	-	0.855	0.3	-	-	-	-	0.652	0.9
Yes	14	45.2	24.0	19.3	29.5	-	-	-	8.0	2.0	12.3	-	-	-	37.0	33.3	39.5	-	-	-
No	15	48.4	33.0	25.5	39.0	-	-	-	8.0	4.5	15.0	-	-	-	35.0	31.0	37.0	-	-	-
Unsure	2	6.5	22.0	17.5	26.5	-	-	-	9.5	6.3	12.8	-	-	-	35.5	34.3	36.8	-	-	-
**Social support**	-	-	-	-	-	-	0.174	5	-	-	-	-	0.375	3.1	-	-	-	-	0.719	1.3
Yes	25	80.7	25.0	20.0	32.0	-	-	-	9.0	3.0	16.0	-	-	-	37.0	33.0	38.0	-	-	-
No	3	9.7	39.0	36.0	39.0	-	-	-	5.0	4.5	7.0	-	-	-	35.0	31.0	36.0	-	-	-
Unsure	2	6.5	27.5	24.8	30.3	-	-	-	9.0	3.5	6.5	-	-	-	35.0	32.0	38.0	-	-	-
**Spending leave days on work-related tasks**	-	-	-	-	-	17.0	< 0.001*	-	-	-	-	42.5	0.025*	-	-	-	-	57.5	0.119	-
Yes	23	74.2	32.0	35.0	39.0	-	-	-	9.0	4.5	16.0	-	-	-	35.0	31.0	37.0	-	-	-
No	8	25.8	18.5	13.0	21.3	-	-	-	3.0	2.0	5.5	-	-	-	38.0	34.5	41.3	-	-	-
**Taking regular holidays**	-	-	-	-	-	35.0	0.003*	-	-	-	-	95	0.683	-	-	-	-	96	0.715	-
Yes	11	35.5	21.0	18.5	26.5	-	-	-	7.0	2.5	14.5	-	-	-	35.0	33.5	37.5	-	-	-
No	19	61.3	32.0	25.5	39.0	-	-	-	8.0	4.0	15.0	-	-	-	35.0.42	31.0	38.0	-	-	-
Unsure	1	3.2	13.0	13.0	13.0	-	-	-	3.0	3.0	3.0	-	-	-	38.0	38.0	38.0	-	-	-
**Experiencing patient volume as reasonable**	-	-	-	-	-	-	0.373	1.9	-	-	-	0.372	1.9	-	-	-	-	-	0.234	2.9
Yes	9	29.0	30.0	21.0	33.0	-	-	-	4.0	2.0	14.0	-	-	-	35.0	33.0	37.0	-	-	-
No	18	58.1	25.5	22.3	32.8	-	-	-	12.5	4.3	12.3	-	-	-	37.0	31.8	38.8	-	-	-
Unsure	4	12.9	38.5	31.8	39.3	-	-	-	4.0	7.5	16.5	-	-	-	35.0	27.0	34.3	-	-	-
**Adequate resources at work**	-	-	-	-	-	-	0.533	2.2	-	-	-	0.566	0.6	-	-	-	-	-	0.396	4.2
Yes	13	41.9	25.0	22.0	33.0	-	-	-	4.0	2.0	16.0	-	-	-	37.0	35.0	40.0	-	-	-
No	12	38.7	30.5	20.5	33.5	-	-	-	8.5	6.5	14.5	-	-	-	33.0	30.8	37.0	-	-	-
Unsure	5	16.1	26.0	25.0	39.0	-	-	-	5.0	4.0	8.0	-	-	-	37.0	37.0	38.0	-	-	-
**Experiencing work environment as pleasant**	-	-	-	-	-	-	0.417	1.7	-	-	-	-	0.661	0.8	-	-	-	-	0.661	3.7
Yes	16	51.2	25.0	19.5	34.3	-	-	-	4.0	2.0	11.5	-	-	-	37.5	34.8	38.5	-	-	-
No	9	29.0	30.0	25.0	33.0	-	-	-	9.0	7.0	13.0	-	-	-	31.0	29.0	35.0	-	-	-
Unsure	6	19.4	33.0	24.5	38.0	-	-	-	10.5	4.3	16.0	-	-	-	37.5	34.0	38.5	-	-	-
**Considering oneself resilient**	-	-	-	-	-	-	0.316	2.3	-	-	-	-	0.130	4.1	-	-	-	-	0.367	2
Yes	27	87.1	26.0	21.5	36.5	-	-	-	7.0	3.0	16.0	-	-	-	37.0	33.0	33.0	-	-	-
No	2	6.5	34.5	32.3	36.8	-	-	-	11.3	10.3	12.8	-	-	-	30.0	28.5	31.5	-	-	-
Unsure	2	6.5	25.0	21.0	29.0	-	-	-	6.0	5.0	7.0	-	-	-	34.0	31.5	31.5	-	-	-
**Good relationship with fellow psychiatric registrars**	-	-	-	-	-	29.0	0.803	-	-	-	-	26.0	0.867	-	-	-	-	2.0	0.030*	
Yes	28	90.3	27.0	21.8	34.3	-	-	-	7.5	3.0	14.5	-	-	-	36.0	33.0	38.0	-	-	-
No	2	6.5	26.0	19.5	32.5	-	-	-	8.0	7.5	8.5	-	-	-	27.5	27.6	27.8	-	-	-
Unsure	1	3.2	35.0	35.0	35.0	-	-	-	20.0	20.0	20.0	-	-	-	37.0	37.0	37.0	-	-	-
**Good relationship with colleagues in other specialities**	-	-	-	-	-	36.5	0.489	-	-	-	-	48.5	0.890	-	-	-	-	54.0	0.604	-
Yes	27	87.1	28.0	21.5	36.5	-	-	-	7.0	3.5	15.0	-	-	-	35.0	32.0	38.0	-	-	-
No	1	3.2	39.0	39.0	39.0	-	-	-	9.0	9.0	9.0	-	-	-	27.0	27.0	27.0	-	-	-
Unsure	3	9.7	26.0	19.5	28.5	-	-	-	8.0	5.5	12.0	-	-	-	38.0	32.0	38.0	-	-	-
**Good working relationship with consultants**	-	-	-	-	-	-	0.086	4.8	-	-	-	-	0.062	5.6	-	-	-	-	0.367	2.0
Yes	27	87.1	37.0	32.0	38.0	-	-	-	7.0	3.0	11.5	-	-	-	37.0	32.0	38.0	-	-	-
No	2	6.5	32.0	29.5	34.5	-	-	-	16.0	12.5	19.5	-	-	-	32.0	29.5	34.5	-	-	-
Unsure	2	6.5	33.0	33.0	33.0	-	-	-	15.0	14.5	15.5	-	-	-	33.0	29.5	33.0	-	-	-
**Training programme offers adequate supervision**	-	-	-	-	-	-	0.094	6.4	-	-	-	-	0.669	1.6	-	-	-	-	0.223	4.4
Yes	11	35.5	25.0	19.5	34.5	-	-	-	5.0	2.0	16.0	-	-	-	37.0	34.5	41.0	-	-	-
No	13	41.9	33.0	28.0	38.0	-	-	-	9.0	7.0	14.0	-	-	-	33.0	30.0	37.0	-	-	-
Unsure	6	19.4	24.5	18.8	25.8	-	-	-	6.0	3.3	9.5	-	-	-	37.5	35.5	38.0	-	-	-
**Psychiatry first choice of specialisation**	-	-	-	-	-	-	0.796	0.5	-	0.7	0.717	-	-	-	-	-	-	-	0.293	2.5
Yes	26	83.9	27.0	21.3	36.8	-	-	-	6.0	3.0	13.8	-	-	-	37.0	33.0	38.0	-	-	-
No	2	6.5	26.0	19.5	32.5	-	-	-	12.5	9.8	15.3	-	-	-	24.5	22.8	26.3	-	-	-
Unsure	3	9.7	33.0	29.0	34.0	-	-	-	6.0	9.0	15.0	-	-	-	34.0	31.5	35.5	-	-	-
**Considering oneself a perfectionist**	-	-	-	-	-	-	0.065	5.5	-	-	-	-	0.427	1.7	-	-	-	-	0.204	3.2
Yes	14	45.2	33.5	26.3	39.0	-	-	-	9.0	3.5	17.0	-	-	-	37.0	33.0	37.8	-	-	-
No	13	41.9	25.0	21.0	31.0	-	-	-	7.0	4.0	13.0	-	-	-	35.8.0	31.0	37.0	-	-	-
Unsure	4	12.9	21.5	16.0	29.5	-	-	-	6.0	3.8	10.0	-	-	-	36	35.8	38.3	-	-	-
**Excessive paperwork**	-	-	-	-	-	-	0.210	3.1	-	-	-	-	0.086	4.9	-	-	-	-	0.416	6.4
Yes	26	83.9	30.0	24.5	37.3	-	-	-	9.0	4.3	16.0	-	-	-	35.0	31.0	37.8	-	-	-
No	3	9.7	18.0	14.0	20.5	-	-	-	2.0	1.5	2.0	-	-	-	38.0	37.5	40.0	-	-	-
Unsure	2	6.5	30.5	26.3	34.8	-	-	-	3.5	2.8	4.3	-	-	-	39.0	38.0	40.0	-	-	-
**Violence as a real threat by patients**	-	-	-	-	-	-	0.217	3.1	-	-	-	-	0.467	1.5	-	-	-	-	0.118	4.3
Yes	18	58.1	31.5	24.5	38.8	-	-	-	8.5	4.3	16.0	-	-	-	34.0	31.0	37.0	-	-	-
No	8	25.8	23.5	19.5	28.5	-	-	-	7.0	2.0	11.5	-	-	-	37.5	34.8	41.3	-	-	-
Unsure	5	16.1	25.0	19.0	30.0	-	-	-	3.0	3.0	8.0	-	-	-	38.0	37.0	40.0	-	-	-
**Impact of patient suicides**	-	-	-	-	-	-	0.188	4.8	-	-	-		0.135	5.6					0.449	2.6
Yes	9	29.0	38.0	23.0	39.0	-	-	-	9.0	5.0	16.0	-	-	-	33.0	29.0	37.0	-	-	-
No	14	45.2	25.0	18.5	29.8	-	-	-	9.0	4.0	16.0	-	-	-	37.5	34.3	38.0	-	-	-
Unsure	5	16.1	30.0	30.0	330	-	-	-	5.0	2.0	8.0	-	-	-	35.0	33.0	37.0	-	-	-
**Experiencing overtime hours as reasonable**	-	-	-	-	-	-	0.791	0.5	-	-	-	-	0.543	1.2	-	-	-	-	0.374	1.9
Yes	24	77.4	27.0	22.8	33.0	-	-	-	8.0	2.8	13.3	-	-	-	35.0	31.0	38.0	-	-	-
No	3	9.7	43.0	32.0	44.5	-	-	-	20.0	12.5	21.5	-	-	-	37.0	36.0	37.5	-	-	-
Unsure	4	12.9	29.0	18.5	39.3	-	-	-	4.5	3.8	7.8	-	-	-	38.0	35.0	41.0	-	-	-
**Sleep deprived**	-	-	-	-	-	84.5	0.063	-	-	-	-	142.5	0.386	-	-	-	-	124.5	0.981	
Yes	22	71.0	31.0	24.3	38.8	-	-	-	7.5	4.0	12.3	-	-	-	36.0	31.5	37.9	-	-	-
No	8	25.8	23.5	18.3	26.0	-	-	-	16.0	13.0	18.0	-	-	-	34.5	31.8	41.3	-	-	-
Unsure	1	3.2	13.0	13.0	13.0	-	-	-	3.0	3.0	3.0	-	-	-	38.0	38.0	38.0	-	-	-
**Debt**	-	-	-	-	-	109.5	0.026*	-	-	-	-	87.0	0.384	-	-	-	-	40.5	0.055	
Yes	5	16.1	35.0	33.0	38.0	-	-	-	9.0	8.0	9.0	-	-	-	31.0	29.0	33.0	-	-	-
No	23	74.2	25.0	20.5	30.5	-	-	-	7.0	2.5	15.0	-	-	-	37.0	33.5	34.5	-	-	-
**Attending therapy**	-	-	-	-	-	31.5	0.043*	-	-	-	-	52.5	0.374	-	-	-	-	67.0	0.914	-
Yes	6	19.4	39.0	32.3	42.0	-	-	-	11.5	6.0	18.5	-	-	-	37.0	34.0	37.8	-	-	-
No	23	74.2	25.0	22.5	32.5	-	-	-	8.0	3.0	14.5	-	-	-	35.0	33.0	38.0	-	-	-
**Using medications to cope with work**	-	-	-	-	-	50.5	0.265	-	-	-	-	70.5	0.938	-	-	-	-	45.5	0.169	-
Yes	6	19.4	34.0	28.5	38.8	-	-	-	9.0	6.0	12.0	-	-	-	33.0	30.8	36.0	-	-	-
No	24	77.4	25.0	20.8	33.0	-	-	-	7.5	3.0	16.0	-	-	-	37.0	33.8	38.3	-	-	-
**Using substances to cope with work**	-	-	-	-	-	29.0	0.474	-	-	-	-	29.5	0.496	-	-	-	-	34.5	0.747	-
Yes	3	9.7	31.0	28.5	34.5	-	-	-	9.0	8.5	12.5	-	-	-	33.0	33.0	35.5	-	-	-
No	26	83.9	26.5	21.3	34.5	-	-	-	7.5	3.0	15.5	-	-	-	36.0	31.5	35.5	-	-	-

EE, emotional exhaustion; DP, depersonalisation; PA, personal accomplishment.

† , In cases where *n* ≠ 31, the remainder of participants chose not to answer the question.

* , Statistically significant.

Utilising leave days for work-related tasks was the only factor associated with higher DP scores, while a good relationship with fellow psychiatric registrars was associated with higher PA scores (Table 5).

## Discussion

### Key findings

A 60.0% response rate is in keeping with average response rates of 52.7% in surveys collected from individuals and used for organisational research.^32^ The possible reasons behind this response rate include registrars being too burnt out to participate in another activity or suspicious regarding confidentiality. The lowest response rate was obtained from registrars > 36 months into registrarship, perhaps indicating that this group did not resonate with the topic or were too overwhelmed with demands to participate.

### Objective 1

Participants scored highest in the EE category, followed by DP and lastly PA. Higher EE and DP scores, and lower PA scores, correlate to greater experienced burnout.^8^ A third of participants had PA scores in the high burnout category. Symptoms of burnout in the EE and DP categories have been hypothesised to serve a purpose, namely, to shield the human psyche against further damage when confronted with having no way out.^1^ Emotional exhaustion was the subscale most affected. Maslach et al. linked the EE subscale to competition, a time-pressured way of life, hostility, a disproportionate need for control and a lack of work–life balance.^8^ Regarding the DP category, Shanafelt et al. showed that DP reflects detachment and an impersonal response to patients;^33^ this may be particularly detrimental in psychiatry where emotional connection and rapport are imperative.

Jovanović et al. found a 60.0% prevalence rate of high burnout among South African psychiatric registrars in 2010 using the DP and EE subscales (second highest in the world after Hong Kong), and this study found a rate of 67.7% using all the three subscales. South Africa was also the country with the second longest working hours after Hong Kong.^11^ In a more recent study among registrars at the WITS School of Clinical Medicine, Zeijlemaker et al. found a prevalence rate of high burnout of 84.0% using the DP and EE subscales, much higher than the rate in the sample.^34^ This may indicate that other specialities have higher rates of burnout than psychiatric registrars. However, the study did not provide specific rates for specialities, and hence, it is a difficult comparison to make.

### Objective 2

None of the demographic or work-related factors were significant predictors of burnout, and this may have been because of the small study sample. Another explanation is that burnout is an organisational complication rather than an individual circumstance.

### Objective 3

Significant factors associated with burnout included poor work and non-professional life balance, using leave days for work-related tasks, not taking regular holidays and financial debt, and are similar to those identified by Fothergill et al.^17^ A significant association was found between participants who experienced EE and who attended psychotherapy. This may speak to the help-seeking behaviour and emotional insight among registrars suffering from EE. In keeping with findings by Fischer et al., a protective factor against burnout was an amicable relationship with fellow registrars in psychiatry.^23^ This emphasises the importance of support rather than competition within the workplace.

### Strengths and limitations

Burnout among doctors is increasingly topical, and studies of this nature form the foundation for planned interventions and, hence, are imperative. However, these results should be interpreted critically, especially with regards to generalisability as the sample size was small. It was a cross-sectional study, which infers that causation cannot be established. The ethics of dual agency should be considered as one of the researchers was a psychiatric registrar at the time of the study;^35^ however, professional integrity and confidentiality were maintained.

### Implications and recommendations

The results of this study may create a platform for further research studies. It is also recommended that this study’s findings influence the university’s policy in terms of sufficient protected academic time (recommended 8h/week according to the HPCSA guidelines) and support.^36^ Future HPCSA guidelines should take into account the phenomenon of burnout and possible interventions, such as counselling. Interventions for burnout should take place at both an individual and institutional level, as a combination of these strategies has been found to be most effective.^37^ Further studies using qualitative methods may provide insights into the unique lived experiences of psychiatrists.

## Conclusion

Evidence of burnout was found in more than two-thirds of WITS psychiatric trainees. Associated factors were found at an organisational level. While optimising individual resilience is important, systemic support plays a key role.
